# Epidemiology and ecology of West Nile virus in sub-Saharan Africa

**DOI:** 10.1186/s13071-018-2998-y

**Published:** 2018-07-13

**Authors:** Waidi F. Sule, Daniel O. Oluwayelu, Luis M. Hernández-Triana, Anthony R. Fooks, Marietjie Venter, Nicholas Johnson

**Affiliations:** 1 Department of Microbiology, Faculty of Basic and Applied Sciences, Osun State University, Osogbo, Osun State Nigeria; 2 Department of Veterinary Microbiology, University of Ibadan, Ibadan, Oyo State Nigeria; 3 Centre for Control and Prevention of Zoonoses, University of Ibadan, Ibadan, Oyo State Nigeria; 4 Animal and Plant Health Agency, Woodham Lane, Addlestone, Surrey KT153NB UK; 5 Department of Clinical Infection, Microbiology and Immunology, Institute of Infection and Global Health, University of Liverpool, Liverpool, UK; 6 Emerging Arbo and Respiratory Program, Centre for Viral Zoonosis, Department of Medical Virology, University of Pretoria, Pretoria, South Africa; 7 Faculty of Health and Medicine, University of Surrey, Guildford, Surrey GU27XH UK

**Keywords:** West Nile virus, Sub-Saharan Africa, Mosquito, Birds, Migration

## Abstract

West Nile virus (WNV) is the aetiological agent of the mosquito-borne zoonotic disease West Nile fever. The virus, first isolated in Uganda in 1937, evolved into two distinct lineages in sub-Saharan Africa (SSA) that subsequently spread to most continents where the virus has evolved further as evident through phylogenetic analysis of extant genomes. Numerous published reports from the past 70 years from countries in SSA indicate that the virus is endemic across the region. However, due in part to the limited availability of diagnostic methods across large areas of the continent, the human burden of WNV is poorly understood. So too are the drivers for translocation of the virus from countries south of the Sahara Desert to North Africa and Europe. Migratory birds are implicated in this translocation although the transient viraemia, measured in days, and the time taken to migrate, measured in weeks, suggest a more complex mechanism is in play. This review considers the evidence for the presence of WNV across SSA and the role of migratory birds in the emergence of the virus in other continents.

## Background

West Nile virus (WNV) is a mosquito-borne flavivirus (family *Flaviviridae*) found throughout the world. Multiple lineages of WNV have been identified based on genomic sequence variation [1]. Two major lineages, lineages 1 and 2, were identified in Africa following the first isolation in Uganda. Lineage 1 occurs mainly in central and northern Africa, Europe, Australia and emerged in the Americas in 1999. Lineage 2 is endemic in southern Africa and Madagascar and emerged in central Europe in 2005. Less common lineages (lineages 3 and 4 in Russia, 5 in India and 6 in Spain) likely evolved from separate introductions into the Northern Hemisphere [2] and further lineages are being discovered in Africa [3]. Lineages 1 and 2 are most important from a public health standpoint causing epidemics in North America and Europe [4, 5] and are likely under-reported in Africa. Phylogenetic analysis, mouse neuroinvasive experiments and clinical data suggest that virulence differences between strains exist that are genotype-specific and not associated with lineage [1].

Infection with WNV develops between 3 and 14 days after the bite of a WNV-infected mosquito, and can persist for a further 3 to 6 days, although severe cases may be biphasic and have symptoms for up to 60 days. Symptoms when they occur include fever, rash, headaches, muscle weakness/pains and joint pains or hepatitis [6]. Fewer than 1% show signs of meningo-encephalitis that include severe headache, flaccid paralysis and occasionally death. A high percentage of patients seeking medical attention reported a prolonged recovery period of up to 60 days [6]. Horses are the most commonly affected domesticated animal, although similar to humans, 80% are asymptomatic. However, of the 20% that develop clinical signs, up to 90% of these are neurological with a mortality rate of 30%. Neurological signs, which may include ataxia and tremors of the face and neck muscles are very common. Some horses have cranial nerve deficits, particularly weakness or paralysis of the face and tongue, which may lead to difficulty in swallowing [7]. In South Africa, lineage 2 WNV is detected most often and considered the main cause of neurological disease in horses [8, 9] with lineage 1 only detected in a single fatal neurologic case in a horse and an aborted fetus over an 8-year surveillance period [10].

West Nile virus was first isolated when Smithburn et al. [11] conducted an epidemiological investigation in Africa during the 1930s, in an attempt to isolate yellow fever virus (YFV) from what was assumed to be an endemic zone extending from the west coast across central Africa into Uganda. Blood was drawn from residents that exhibited illness suggestive of YFV infection. One sample was taken from a febrile 37-year-old woman from Omogo, West Nile district, in the Northern Province of Uganda. Serum was inoculated intracerebrally into ten mice, of which only one survived. Sub-inoculations were conducted and in its 53rd passage (September 15, 1939), no surviving mice had been reported since the first mouse passage. Subsequent experiments demonstrated pathogenesis in mice when inoculated intracerebrally, intranasally and intraperitoneally with reduced virulence observed following subcutaneous inoculation. Intracerebral inoculation into Rhesus monkeys resulted in fatal encephalitis. Serum raised against the virus cross-neutralized Japanese encephalitis virus (JEV) suggesting relatedness between the two viruses and grouping WNV in the JEV serocomplex. The lesions induced by the new virus were limited to the central nervous system. The virus was subsequently named after the West Nile District in Uganda [11].

In Europe and the USA, WNV infection in birds have been described with disease presentation manifesting as weight loss, decreased activity, depression, and neurological signs such as torticollis, opisthotonus and rhythmic side-to-side head movements [12]. Following the introduction of WNV into North America, a range of avian species were affected, but particularly members of the family Corvidae such as the common crow (*Corvus brachyrhynchos*) [13]. Goshawks (*Accipiter gentilis*) in central Europe are often reported with fatal WNV infection [12]. In Africa, there have been no descriptions of WNV infection causing disease in African birds although the virus has been isolated from egrets (genus *Egretta*) and indigenous parrots (*Coracopsis vasa*) in Madagascar [14, 15].

**Fig. 1:**
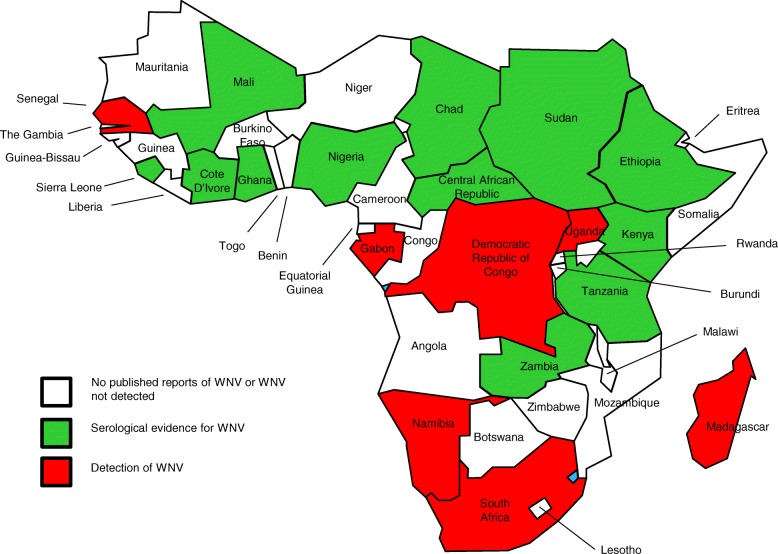
Countries of sub-Saharan Africa showing those where WNV has been isolated (red), those where WNV has been detected by serosurveillance (green) and those where no evidence for WNV has been found or no studies have been reported (white)

West Nile virus has emerged in both Europe and the Americas as a major public health threat in the past two decades. Prior to this, the virus was considered a minor pathogen of humans in the 44 countries that make up SSA (Fig. 1). Bird migration is often cited as the route for the virus to be transported from southern Africa to Europe and beyond [16]. However, few studies or reviews have considered the presence of the virus in SSA, its public health burden or the factors that lead to its spillover to other regions of the world.

## Arthropod vectors of WNV in Africa

West Nile virus is primarily spread when a mosquito is exposed to the virus by feeding on birds that develop high levels of the virus in the blood. Competent infected mosquitoes may transmit the virus if they feed on susceptible vertebrates, including humans [17]. The abundance and feeding patterns of infected mosquitoes, as well as human behavior that influence their exposure to mosquitoes, influence the likelihood of WNV transmission [18]. As a result of the abundance of human biting mosquitoes in SSA, in contrast to the low incidence reported during epidemics in Europe and North America, up to 55% of human populations seroconvert during epidemics in Africa [19, 20].

Experimental transmission of WNV was first documented in *Aedes albopictus* in 1942 [21]. By 1950, experimental transmission was also reported in *Culex pipiens* and *Cx. tritaeniorhynchus*, two species that are found across large areas of Africa. Experimental trials in conjunction with field studies from 1952–1954 in the Sindbis district of Egypt demonstrated that mosquitoes were the vectors of WNV. Isolates were obtained during this study from *Cx. antennatus*, *Cx. univittatus* and *Cx. pipiens* [22]. In Egypt, Israel and South Africa, *Cx. univittatus* has been implicated as the primary WNV vector based on field isolation rates. In Egypt, nine isolates from mosquitoes were obtained at the same time as isolations from febrile children [20]. South African isolates have also been obtained from *Cx. theileri*, *Cx. pipiens*, *Cx. neavei*, *Ae. caballus*, *Ae. circumluteolus* and *Coquillettidia* spp. West Nile virus was isolated in Africa from *Cx. poicilipes* in Senegal and *Ae. albocephalus* in Madagascar [21]. In Nigeria, however, WNV was not isolated from collected *Cx. quinquefaciatus* and *Mansonia* spp. but the former species yielded WNV-specific RNA following RT-PCR [23]. Miller et al. [24] reported isolation of WNV from a male *Cx. univittatus* mosquito trapped in northwestern Kenya, which suggested that WNV can be transmitted transovarially.

Ticks have also been implicated as vectors of WNV, although their importance is unclear. Soft ticks such as *Argas hermanni* and *Ornithodoros capensis* from Egypt and Azerbaijan, respectively, have yielded WNV isolates [21]. Additionally, WNV has been isolated from hard ticks of the genera *Hyalomma*, *Rhipicephalus*, *Amblyomma* and *Dermacentor* in Africa [25], and regions such as Astrakhan, Azerbaijan, Turkmenistan and Moldova [21]. Experimental transmission has been reported in *Argas hermanni*, *Ornithodoros capensis* and *Dermacentor marginatus*.

## Association of WNV with birds

Preliminary research indicated that domestic livestock did not produce sufficient viraemia to infect feeding mosquitoes, a necessity for widespread infection in endemic WNV regions [26]. This led to the suspicion that wild birds serve as reservoirs for WNV. The first evidence for this came from the isolation of WNV from brain, spleen and blood of a pigeon in 1953 from the north-central area of the Nile Delta [27–29]. A number of wild birds have subsequently been documented as reservoir hosts in endemic areas; infected reservoir birds develop transient high-titre viraemia that allows transmission of WNV to feeding mosquitoes [30]. Ecological studies demonstrated that the presence of seropositive humans correlated with the presence of seropositive crows in Egypt in 1955 [27]. In one of the earliest surveys conducted in the mid-1950s in Egypt involving birds, neutralizing antibodies were found in all common avian species tested from endemic areas [27]. Hooded crows (*Corvus cornix*) had the highest prevalence of neutralizing antibodies, especially in endemic WNV areas, as well as a 100% mortality rate in experimentally infected crows. These authors speculated that this could imply a high mortality in naturally infected crows. However, many survived natural infection as indicated by the high antibody prevalence. The authors also found that hooded crows and sparrows (*Passer domesticus*) had sufficient viraemia to complete a bird-mosquito-bird transmission cycle. They were also able to infect birds with WNV-infected *Cx. pipiens* and *Cx. univittatus* mosquitoes [27–29]. In another study involving wild South African birds, 13 species from five different families developed viraemia capable of infecting mosquitoes; infected birds remained viraemic for four days [31].

Migratory birds have been incriminated as the mechanism for WNV introduction, re-introduction, and spread across Europe [32–35] and the Americas [36, 37]. West Nile virus is taken up by a competent ornithophilic mosquito vector during blood-feeding on an infected bird [38]. After a short period necessary for the replication of the virus, the infected mosquito spreads WNV *via* biting reservoir or amplifying hosts (either migratory or indigenous birds), and horses or humans, which are “incidental reproductive dead-end” hosts [39].

In South Africa, Jupp [14] reported that WNV is maintained in an enzootic transmission between feral avians and ornithophilic *Cx. univittatus* in the temperate inland plateau region. About 30 species of birds were reportedly involved in the cycle without WNV-mediated mortality, indicating they are real reservoirs of WNV in the region.

## WNV and other species

Experimental infections have shown that domestic animals rarely develop viral titers or clinical signs after being exposed to WNV. Sheep that were fed on by WNV-infected mosquitoes failed to become viraemic, although one sheep developed neutralizing antibodies implying a productive asymptomatic infection [29]. Experimental studies with pigs showed they are poor hosts for WNV and experimental infection of calves failed to produce a viraemia. In the Highveld Region of South Africa, experimental infection of dogs resulted in a detectable WNV viraemia [14]. Dogs inoculated with WNV subcutaneously and intravenously developed haemagglutination inhibition (HI) and neutralization test (NT) antibodies; one of the inoculated dogs developed a low titer viraemia [29, 40]. Other domestic animals can develop WNV antibodies in natural settings. In Madagascar, 33% of the oxen tested in the town of Mandoto and 2% of the oxen tested in the city of Tsiroanomandidy were seropositive for WNV [29]. A survey of dogs in the Orange Free State, South Africa, revealed that 46% of dogs sampled had HI antibodies against WNV, and the virus was isolated from one of the dogs that was HI negative [14, 40]. Three horses experimentally infected with lineage 2 strain in South Africa did not develop clinical signs and a serosurvey in racehorses showed that 11% of yearlings seroconverted in the first year while 75% of their dams had antibodies [41]. An 8-year surveillance study indicated that 2–13% of neurological cases in horses per annum in South Africa were due to WNV lineage 2 with a 30% mortality rate. In this study, WNV was also detected in one case in a locally bred Ayrshire cow (*Bos taurus*) and a Boer goat (*Capra aegagrus hircus*) with neurological signs suggesting that locally bred livestock may also be affected but are not under the same close surveillance as horses [10].

## Detection and surveillance for WNV infection in Africa

Many areas of SSA are endemic for malaria and typhoid fevers [42]. Hence, cases of febrile illness are usually regarded by physicians as one of these infections [43, 44]. In addition, when malaria and typhoid tests are negative for a given patient, such cases are commonly regarded as “undifferentiated febrile illness (UFI)” or “fever of unknown origin (FUO)”, especially when the patient fails to respond to anti-malarial drugs [45]. Most health facilities lack the capacity to conduct diagnostics for arboviral infections on such UFI patients, and physicians are restricted to treatment based on symptoms.

Clinical signs of WN disease are non-specific and can mimic those caused by other pathogens and toxins; infection in humans or animals should be confirmed through the identification of WNV genome, antigen or specific antibodies. Many tests have therefore been developed for diagnosis and these include WNV isolation in Vero or mosquito cell lines, or isolation in infected suckling mice brain tissue followed by serologic identification; for example, the isolation of WNV from brain biopsies of children who died of encephalitis in India [46]. WNV-specific antibodies as well as antigen can be detected using IgM antibody-capture enzyme-linked immunosorbent assay (MAC-ELISA), HI antibody detection, complement fixation test (CFT), immunofluorescence assay (IFA), microsphere immunoassay and plaque reduction neutralization test (PRNT) [47, 48]. Due to cross-reactions between flaviviruses it is important that serological assays be confirmed by NT. In Africa, many of these methods have been used in surveillance for WNV; a serosurvey of camels, goats, cattle and sheep in Nigeria was conducted by detection of WNV HI antibodies [45]. Additionally, ELISA and PRNT have been used for WNV sero-surveillance in Nigeria [23, 49]. In South Africa, Venter et al. [10] diagnosed WN disease in horses using a combination of post-mortem histopathologic examination of brain samples and WNV-NS5-specific nested real-time RT-PCR of viral RNA extracted from brain sections, which was confirmed by nucleotide sequencing.

## WNV in sub-Saharan Africa

Table 1 provides evidence for the presence or absence of WNV in many of the countries of SSA. This is principally through seroprevalence studies conducted on human populations during the 1970s and 1980s [50–54] and, to a lesser extent, domestic animals [55–57]. Large outbreaks of West Nile fever are occasionally reported in human populations [14, 58, 59] but are rare, go undiagnosed or are blamed on other viral causes of febrile disease such as yellow fever or dengue. One of the largest serological surveys focusing on 2320 humans among the rural populations in Gabon reported an overall WNV-specific IgG prevalence of 27.2%, which was considered high [60]. The prevalence varied according to the ecosystem: 23.7% in forested regions, 21.8% in savannah and 64.9% in the lakes region. The WNV-specific IgG prevalence rate was 30% in males and 24.6% in females, and increased with age. The authors reported that although serological cross-reactions between flaviviruses were likely and might be frequent, their findings strongly suggested that WNV was widespread in Gabon. The difference they observed in WNV prevalence among ecosystems suggests preferential circulation in the lakes region; in addition, the increase in seroprevalence with age suggested continuous exposure of Gabonese populations to WNV. The authors recommended further investigations to determine the WNV cycle and transmission patterns in Gabon [60]. In South Africa, serosurveys conducted in the 1970s indicated differences between the temperate central Highveld region with approximately 7% seropositive humans, the semi-desert Karoo in the Western Cape with 17%, and the coastal region of KwaZulu Natal that had 2% sero-positivity, which is possibly associated with the spread of vectors due to altitude and rainfall. The largest outbreak ever recorded of WN fever occurred in 1974 in the Karoo that included tens of thousands of cases. Another epizootic was reported in the 1980s that included a thousand cases in Gauteng [14]. Since then 5–15 cases are reported annually by the National Institute for Communicable Diseases through passive submission of suspected arbovirus cases for diagnoses although it is thought to be under-reported [61]. Screening of human cerebrospinal fluid (CSF) specimens in hospitals in the Tshwane region (Pretoria) in South Africa using RT-PCR, IgM ELISA and TCID_50_ neutralization assays in 2009 detected WNV in 3.7% of unsolved cases of neurological disease in patients [62]. A serosurvey of veterinarians from across South Africa reported 7.8% seropositivity in 2011–2012 [63].

**Table 1:** Investigations for the presence of WNV in sub-Saharan Africa. The order of countries reflects those where WNV has been detected through virus isolation, those where WNV has been detected through serosurveillance and those where WNV has not been detected or no studies have been reported

Country	Area (km^*2*^)^a^	Population estimate for 2015 (millions)^b^	Reference	Observation
Countries where WNV has been detected through virus isolation				
Democratic Republic of the Congo	2,344,858	71.2	[58]	WNV outbreak in an army camp
Djibouti	23,200	0.9	[82]	Detection of WNV in *Culex* mosquitoes
Gabon	267,667	1.7	[83]	Human case of WNV
Madagascar	587,041	24.2	[15, 84–86]	WNV detected in birds, mosquitoes and humans. Serological evidence for WNV in lemurs and horses
Namibia	824,292	2.4	[87]	WNV detected in humans
Senegal	196,722	15.0	[3, 88]	Serological evidence for WNV in humans. Detection of multiple lineages of WNV
South Africa	1,219,090	53.5	[10, 14, 62]	Serological evidence for WNV in humans, detection in mosquitoes, disease in horses; neurological disease in humans
Uganda	241,038	40.1	[11]	Isolation of virus from humans
Countries where WNV has been detected by serology only				
Central African Republic	622,984	4.8	[53]	Serological evidence for WNV
Chad	1,284,000	13.6	[56]	High seroprevalence among horses
Côte d'Ivoire	322,463	21.3	[55]	High seroprevalence among horses
Ethiopia	1,104,300	98.9	[52]	Serological evidence of WNV
Ghana	238,533	26.4	[89]	Serological evidence for WNV in humans
Kenya	580,367	46.7	[50]	Serological evidence for WNV in humans
Mali	1,240,192	16.2	[90]	Serological evidence for WNV in humans
Nigeria	923,768	182	[57, 91]	Serological evidence for WNV in humans and horses
Sierra Leone	71,740	6.3	[92]	Serological evidence for WNV in humans
Sudan	1,861,484	39.6	[52]	Serological evidence for WNV in humans
Tanzania	947,300	52.3	[51]	Serological evidence for WNV in humans
Zambia	752,618	15.5	[93]	Serological evidence for WNV in humans
Countries that have not reported the presence of WNV				
Angola	1,246,700	22.8	na	–
Benin	112,622	10.9	na	–
Botswana	581,730	2.0	na	–
Burkina Faso	274,200	17.9	na	–
Burundi	27,830	10.8	[54]	No evidence for WNV found
Cameroon	475,440	23.4	[94]	No evidence for WNV found
Congo	342,000	4.7	na	–
Eritrea	117,600	6.7	na	–
Equatorial Guinea	28,051	0.8	na	–
Guinea	245,857	12.3	na	–
Guinea-Bissau	36,125	1.8	na	–
Lesotho	30,355	2.1	na	–
Liberia	111,369	4.5	na	–
Malawi	118,484	17.3	na	–
Mauritania	1,030,700	4.1	na	–
Mozambique	799,380	27.1	na	–
Niger	1,267,000	19.3	na	–
Rwanda	26,338	12.4	na	–
Somalia	637,657	11.1	na	–
South Sudan	644,329	12.1	na	–
Swaziland	17,364	1.3	na	–
The Gambia	11,295	2.0	na	–
Togo	56,785	7.2	na	–
Zimbabwe	390,757	15.0	na	–

*Abbreviation*: na, not applicable (no evidence found)

^a^[95]

^b^[96]

Few seroprevalence studies on WNV were reported during the 20th century in Nigeria. In 1990, Omilabu et al. [64] reported a survey in which CFT was used to detect WNV-specific antibodies in humans and domestic animals. The overall complement fixing antibody to WNV in the two localities surveyed was 65%. Of 170 persons tested, 53 and 75% were positive in Ibadan and Ogbomoso, respectively. WNV antibody prevalence increased with age in both communities. Gender-specific WNV prevalence rates were 49/82 (60%) for females and 62/88 (75%) for males. Tests on animal sera showed that 33% contained CF antibody to WNV; the prevalence rates of CF WNV antibody by animal species were 62% in camels, 4% in cattle and 0% in goats. A parallel serosurvey, using HI assay for WNV antibody, was carried out in humans and domestic animals in Nigeria. Out of 304 human sera tested, 123 were positive (40%). There was a higher prevalence of HI antibody in adults than children. Seroprevalence levels of 37% in males and 43% in females by WNV HI antibody were observed; there was, however, no significant difference in the prevalence rates of HI antibody in both sexes. Of the 123 WNV HI-positive sera tested, 104 (85%) and 78 (75%) had YFV and Potiskum virus HI antibodies, respectively. Monotypic WNV reactions were frequently found in children, while polytypic reactions were predominantly found in adults. The study also tested a total of 200 animal sera: 50 camels, 50 goats, 49 cattle and 51 sheep. The highest prevalence of WNV HI antibody was found in camels (26%), followed by sheep (20%). Percentages of positive sera in other species were: goat (18%) and cattle (6%). Of the 35 WNV HI-positive animal sera, 26 and 20% reacted with yellow fever and Potiskum virus antigens, respectively [48]. Cross-reactions with other flaviviruses cannot, however, be ruled out and future surveys should be confirmed by WNV-specific neutralization assays.

In recent years, more seroprevalence studies have been reported. An ELISA-based WNV antibody prevalence study was conducted by Baba et al. [43], in which sera from 973 febrile patients suspected of malaria and typhoid fever from a semi-arid zone (Sahel savannah zone) were tested for WNV and YFV IgM and IgG by antibody-capture ELISA. Prevalence rates of 1.2% for anti-WNV IgM and 80.16% for WNV IgG were documented. They observed that two serum samples with mixed infections of DENV-2 and WNV detected by MAC-ELISA also had neutralizing antibodies against WNV. They also reported overall concordance between the PRNT and ELISA results. According to the study, the high prevalence of WNV IgG obtained indicated endemicity of WNV in semi-arid zones of Nigeria. The prevalence of WNV IgM and IgG antibodies as well as the ages and gender of the patients were not notably different. The authors advised confirmation of WNV IgM-positive sera with PRNT and that the ELISA and PRNT should be complemented with reverse transcriptase-polymerase chain reaction (RT-PCR) when the time of onset of illness was not considered in sample collection. They further suggested a need to include arboviruses in the differential diagnosis of febrile illnesses in Nigeria.

In Osun state, southwestern Nigeria, Opaleye et al. [49] reported no serological evidence of WNV infection in blood donors. Regarding WNV in animals, neutralizing anti-WNV antibodies have been documented in horses, donkeys and camels in Borno State, Nigeria [65] while Sule et al. [57] demonstrated WNV seroprevalence in polo ponies in southwestern Nigeria. In addition, there is documented evidence for the involvement of WNV in FUO in Nigeria [43]. In another study, it was reported that 45.0% (18/40) of febrile participants had WNV antibodies but were negative for malaria parasite and Widal tests, thereby accounting for UFI [66].

Increasingly, surveys suggest a high seroprevalence of WNV antibodies in Nigerian populations. This in turn indicates that WNV is highly endemic within Nigeria and further investigations for the presence and distribution of virus in reservoir avian species and vector mosquito species are needed.

## West Nile virus in North Africa

The epidemiology of WNV in Northern Africa has recently been reviewed [67] and it is only briefly discussed below. In Algeria, an epidemic occurred between August and September 1994 in the oasis town of Timimoun in central Sahara. About 50 individuals presented with high fever and neurological signs; 20 were clinical cases of encephalitis, of which 8 died. WNV serology on 18 cases (14 clinical cases and 4 probable) revealed 17 to be positive for the virus. All the 14 clinical cases were IgM-positive and 13 were children 10 months to 9 years old [68]. Between August and mid-October 1996, 94 equines were affected in Morocco (in the provinces of Kenitra and Larache), 42 of which died, and the disease was reported in all age categories [69]. The virus was isolated from a brain biopsy. A human encephalitis case was also suspected to be due to WNV. The Ministry of Agriculture reported nine equine WNV cases (including five fatal cases) that occurred in September 2003 in Kenitra province. In 2012, El Rhaffouli et al. [70] reported that of the 499 participants they studied, 59 (11.8%) had WNV neutralizing antibodies (7 of 150 in cohort A, 24 of 200 in cohort B, and 28 of 149 in cohort C). They found evidence for local circulation of WNV in Morocco and concluded that Moroccan WNV strains most often cause mild and self-limiting illnesses. The illnesses were reported to be difficult to distinguish from many other febrile illnesses, making it less likely that viral testing would be performed for WNV. In Tunisia, 173 patients were hospitalized for meningitis or meningo-encephalitis in two coastal districts, Sfax and Mahdia, between September 7 and December 12, 1997, and 8 died. The epidemic peak was reached between the last week of October and the second week of November. Among 129 patients tested, 111 cases were WNV IgM ELISA-positive (86%) including five fatal cases (4.5%). Of the positive cases, WNV IgM ELISA was performed on the CSF of 23 patients and was positive for 9 cases including 6 (3 fatal cases) for whom only CSF could be obtained [29]. The virus was also isolated from brain tissue samples.

## Conclusions

The cumulative evidence from seroprevalence studies, outbreak reports and virus isolations suggest that WNV is endemic throughout SSA wherever susceptible mosquito populations are abundant. The cycling of virus between mosquitoes and birds provides a reservoir for the virus that can then be transmitted to dead-end hosts such as humans and horses. Despite evidence for virus isolation from a range of mosquito species, there are few systematic studies in either mosquito or bird populations in recent years that confirm the existence of this cycle, or identification of factors leading to spill-over into the human population. There is a strong need to protect human populations in Africa from mosquito-borne diseases such as malaria, yellow fever, dengue fever and WN fever. However, these preventive strategies rely on low-tech options that aim to prevent mosquito bites. Protection can be achieved by avoiding areas where mosquitoes are active, avoiding outdoor activities during peak mosquito biting times at dawn, dusk and early evening, and when outdoors by applying mosquito repellent to clothing and exposed skin. Reduction of mosquito breeding sites by regularly emptying water from flower pots, pet food and water dishes, bird-baths, swimming pool covers, buckets, barrels, and cans is a simple measure to reduce mosquito populations close to human habitations. In regions with high rainfall, unblocking rain gutters and removing discarded vehicle tyres and other items that could collect water also help to reduce breeding sites. Although there is a WNV vaccine licensed for horses, there is no equivalent for humans [71, 72] and no effective antiviral prophylaxis. Therefore, surveillance offers one option for monitoring for the emergence of disease in human populations. Barnard & Voges [73] reported a positive correlation between occurrence of symptomatic equine and human cases caused by *Flavivirus* infection, suggesting that equine outbreaks might predict disease risk for humans. So, as part of prevention strategies, it has been advocated that horses could be used as sentinels for detecting WNV activity and emergence of virulent strains [10]. Phylogenetic studies suggest that WNV evolved in SSA and that recent emergence of the virus in Europe has an origin in Africa [74], although only four known lineages have been reported from the continent. The hypothesis that WNV is translocated from SSA to northern latitudes by migratory birds could explain the repeated emergence in Europe [75]. This is supported by studies reporting the isolation of virus in migratory birds from Israel [76] and Slovakia [77]. However, since the time required to cover the distances involved is measured in weeks and not days, it is unlikely that a bird that is viraemic for a few days, leaving SSA, will remain infectious to a biting mosquito in Europe. A recent study failed to detect flaviviruses in migratory birds entering Italy [78] although this does not exclude the possibility that it happens. If migratory birds leaving SSA play a role in WNV translocation, it is likely to be through relatively short distance movements between resting grounds as they journey north. Sites around the Mediterranean Sea where migrating birds rest and mosquito vectors are abundant are considered “hotspots” for WNV introduction into Europe [79]. This likely extends further south to locations such as the Nile Valley and oases within the Sahara Desert where birds rest. It is therefore unsurprising that these locations have experienced outbreaks of WNV in the past [27, 68]. Detection of WNV in ticks in a number of locations across SSA may also suggest a potential source for infection if carried by migratory birds into Europe although the susceptibility of ticks as transmission vectors needs further investigation. It is likely that similar locations where birds congregate exist throughout Africa and the Middle East providing suitable conditions for localised transmission of WNV that in turn contributes to long-distance translocation of virus. The presence of WNV in North Africa [80] and the Middle East [81] suggests that such a scenario is possible.

## Abbreviations

CFT: Complement fixation test
CSF: Cerebrospinal fluid
FUO: Fever of unknown origin
HI: Haemagglutination inhibition
JEV: Japanese encephalitis virus
MAC-ELISA: IgM antibody capture enzyme-linked immunosorbent assay
na: Not applicable
NT: Neutralization test
PRNT: Plaque reduction neutralization test
RNA: Ribonucleic acid
RT-PCR: Reverse transcriptase polymerase chain reaction
SSA: Sub-Saharan Africa
TCID: Tissue culture infectious dose
UFI: Undifferentiated febrile illness
WNV: West Nile virus
YFV: Yellow fever virus
